# Medical and Physical Intelligence

**Published:** 1824-12

**Authors:** 


### MEDICAL AND PHYSICAL INTELLIGENCE.

### PHYSIOLOGY.

1. *Experiments on Vomiting.*—PROFESSOR TANTINI has lately performed a variety of experiments, with a view of ascertaining the degree of confidence to be placed in the opinions of M. MAGENDIE upon this subject. He states that, when he left the cardia free, in substituting a bladder for the stomach, after the manner done by the French physiologist, no vomiting resulted; but, when he introduced into the cardiac orifice the cannula which served to connect the bladder which was introduced in the place of the stomach, vomiting immediately took place. From this he concludes, that, in the act of vomiting, we must take into the account, not only the contraction of the abdominal muscles and of the diaphragm, but likewise of the muscular coat of the stomach itself.—(*Annali Universali,* July 1824.)

2. *Wasting of the Optic Nerves with Blindness.*—We quote this case to prove (if proofs be wanting,) that the position of M. MAGENDIE concerning the fifth pair as the nerves of vision, is untenable. M.

Leveillé found a tumor, about the size of a hen's egg, between the dura mater and the arachnoid, at the anterior and external part of the left lobe of the cerebrum, the convolutions of which were effaced to a great extent. The individual who presented this phenomenon had been long in a state of complete imbecility, without offering any other peculiarity. He had been blind for two years, and both of the optic nerves were entirely wasted, *(entièrement atrophiés.)*—*(Revue Med.* Sept.)

---

### PATHOLOGY.

3. *Death from the Introduction of Air into the Veins during a Surgical Operation.*—At a recent meeting of the Academie Royale de Medecine, M. Samson read, in the name of M. Dupuytren, the following account:—A young girl, named Poirier, remarkable for her beauty, and the strength of her constitution, entered the Hôtel Dieu, on account of a tumor which occupied the posterior and lateral part of the neck. From its hardness, its virulence, and its want of sensibility, M. Dupuytren easily recognised that it was of a fibrous nature, and determined on removing it before it had acquired a larger size. The operation was conducted with much skill and rapidity. The tumor no longer adhered, except to the anterior shred of the integuments; and the patient, who had only lost a very small quantity of blood, bore without much complaint the pain inseparable from so minute a dissection, when all at once a hissing noise of some duration *(sifflement prolongé)* was heard, analogous to that which results from the entrance of air into an exhausted receiver. The operator stopped for a moment in astonishment. "If I was not so far from the air-passages," said he, "I should think I had opened them." Scarcely had he finished the sentence, and given the last cut which was required to separate the tumor, when the patient exclaimed "I am dead!" She was immediately seized with general tremor, then fainted in the chair, and fell motionless: in vain was every means employed—the girl had ceased to live.

On opening the body, the pericardium was found to be perfectly healthy; the right auricle of the heart was distended with air, which gave it an elastic tension, and, when its parietes were cut, this air escaped in great part, without any admixture of blood: this cavity, however, contained a small quantity of uncoagulated blood. Some blood, likewise fluid, was found in the other cavities of the heart, which were sound, and in the arteries and veins of the body, the limbs, and the brain; and, so much air was there, that the vessels, on being pricked at different points, every where gave vent to bubbles mingled with blood. The other organs presented nothing remarkable.

It is, then, to the introduction of a considerable quantity of air into the heart, that death is to be attributed. The manner in which this introduction took place is easily ascertained: a vein of considerable size, situated at the lower part of the tumor, and communicating with the jugular, was necessarily opened, and, continuing to gape at the moment when the act of inspiration drew the blood towards the chest, was filled with air, which the blood, and a movement of the tumor, must have thrown upon the heart. The air, becoming rarified in the cavities of this organ, had distended them, prevented their contraction, and thus suddenly produced syncope and death.—(*Revue Medicale*, Sept.)

4. *Pustules under the Tongue in Hydrophobia.*—Professor KOREFF has addressed a letter on this subject to Baron DUPUYTREN, which is too important to pass over in silence. The observations of M. MAROCHETTI are already well known to the profession: he tells us that the hydrophobic virus is to be found in the orifices of the sublingual glands, on each side of the frœnum of the tongue, where one or more pustules, about the size of a lentil or millet-seed, are formed, evidently containing a fluid, which after a certain time is re-absorbed, and then the symptoms of hydrophobia burst forth; and this occurs unless the virus is destroyed within twenty-four hours after its formation. M. Koreff believes that the popular superstition of looking for a worm under the tongue in dogs, is connected with some former knowledge of the above fact; and calls to our notice a dissertation published in 1777 by HEYSHAM, in which he asserts that this worm under the tongue is merely a gland destined to the secretion of the hydrophobic virus.

Since the publication of M. Marochetti's work, M. REHMANN, of Petersburg, has observed these pustules under the tongue of a man who died of hydrophobia; but the matter contained in them was found quite indurated.

Professor ERDMANN has published an account of a method analogous to that described by M. Marochetti, employed in Esthonia, at least four hundred leagues from the spot where that gentleman made his discovery. The operator in this case was a peasant; his art was not his own invention, but transmitted by tradition; his method of treatment did not differ essentially from M. Marochetti's. Several medical men then in Prussia, in consequence of the circular order issued by the minister, have confirmed the existence of these pustules; particularly Dr. BAUMBACH, at Erfurt, who, by opening the pustules, and treating them as directed, saved his patient. Drs. ETMULLER and IDELER were not fortunate enough to save their patient, a man of sixty years of age, but they evidently were called in too late: they, however, found four pustules under the tongue.

M. Koreff observes, that so many witnesses must put this fact beyond all reasonable doubt: nor must it be looked upon as accidental, even should it not invariably be found to take place, since many circumstances may prevent the development of the pustules. If, in the first instance, the virus has been destroyed by cauterization or otherwise, they will not be found; or where the venom of the animal is exhausted, as may be the case if many persons have been bitten in succession. There may also be persons not susceptible of this poison; as is the case with regard to syphilis, variola, and plague, in certain individuals. Nor ought we to pronounce this discovery to be an illusion, as some have done, because the pustules cannot always be found when hydrophobia exists; since it is admitted that their appearance is but transitory, and that they do not, in general, last above twenty-four hours. There is some reason to think (as in the cases published by M. MAGISTEL,) that an inefficient cauterization of the bites is likely to hasten the appearance of these pustules. M. Koreff finally remarks, that the decoction of the genista can only be looked upon as a secondary means of cure, after the pustules have been opened and cauterized, and the mouth has been gargled with a strong decoction of the same plant.—(*Journal Comp.* August.)

We conceive that the above abridged account of M. Koreff's letter will be sufficient to draw the attention of practitioners to this interesting point, and that no opportunity will in future be neglected of daily examining the tongue in those cases where the inoculation of hydrophobia may be suspected.—EDITORS.

5. *New Species of Acaris living on the Human Body.*—M. BORY DE ST. VINCENT relates the following case:—A woman, forty years of age, who had been an invalid upwards of twenty years, having obtained no relief from medicine, placed herself under the care of a person who pretended, by the use of a violent remedy, to restore her to health. She did experience sensible relief; but at the same time violent itching began to be felt over the whole body, and thousands of little brownish-coloured animals, almost imperceptible, made their appearance from those parts of the body which she had scratched. M. St. Vincent examined them with a microscope that magnified five hundred times, and found them to be a new species of acaris, much resembling the *ixodes*, having a small sucker and two feelers, composed of four joints each. The woman did not communicate these insects to any other person, not even to her husband, with whom she continued to sleep. The patient soon relapsed and died.—(*Journal Comp.* August.)

6. *Rupture of the Vena Cava.*—M. DOMMANGET relates the case of an officer of light infantry, of the army of Spain, aged twenty-five, who had enjoyed general good health until a month prior to the 23d of December, 1822, when he was confined to his house by a catarrhal affection, followed by an inflammation of the pleura on the right side. An antiphlogistic treatment removed these affections; but, on the 25th, the patient experiencing a dull pain between the shoulders, two cupping glasses were applied, and the pain disappeared. He continued to get better, and on the 30th was completely convalescent, passing the day with his comrades, and going to bed apparently in good health. On the 31st, at nine o'clock in the morning, the patient complained suddenly of pain resembling the colic, situated just below the pit of the stomach, with a sensation of weight at the epigastric region, which he supposed to be occasioned by the weight of something which he could not get rid of, notwithstanding the frequent endeavours to vomit which he experienced. When seen by Dr. BERGE (in the absence of M. Dommanget), he was found sitting upon his bed, his body leaning forwards, the hands crossed upon the epigastric region, which he was pressing forcibly, complaining of deep-seated and very violent pains; the abdomen retracted; the face pale and pinched in; respiration laboured, and a small, but not frequent, pulse. The patient lived in this condition about two hours, making repeated efforts to vomit, the respiration becoming gradually quicker, and the sense of suffocation more intense.—Dissection showed at once the cause of this officer's death. The lungs were in a healthy condition, but at the lower part, on the right side, a quantity of coagulum, weighing thirty-six ounces, was found, and at least twelve ounces of bloody serum; on the left side another coagulum, weighing two ounces, was found, and the lung on this side had contracted, at its upper part, some slight adhesions to the pleura costalis. On examining, a tumor of two inches and a half in width, by five inches in length, was found on the right side of the vertebral column, in the course of the vena cava: towards the middle of this tumor were several abrasions, or ragged openings of the pleura, about half an inch in length, from which the blood had escaped into the chest. On dissecting the tumor carefully, it presented the appearance of a substance analogous to the spleen, though not quite so firm: fibres, running in every direction, were observed. In the centre of this fungous tumor, which appeared to be a recent formation, there was a rent parallel to that in the pleura, corresponding to a canal ten lines in diameter, which formed a substitute for the vena cava for the space of about four inches and a half, extending to the right auricle. Two other tumors, resembling the former, were found,—one in the left side of the chest, the other on the small curvature of the stomach, near the cardiac extremity. The author concludes the above tumor to have been of the nature of fungus hæmatodes.—(*Journal General,* August.)

7. *Rupture of the Heart.*—M. BAYLE relates the case of a lady, aged sixty-eight, corpulent, who in the time of the Revolution had experienced a great reverse of fortune, but who had for a long time been restored to comfort, and had enjoyed good health. On the 7th of last June, having a slight degree of fever, she consulted her physician, who found her with a slight cough and difficulty of breathing, the pulse not much accelerated and quite regular; the chest sounded well on percussion throughout, and the contractions of the heart were natural; she was rather constipated; and had been accustomed yearly, every spring, to catarrhal affections, which usually lasted about a week. She also said that she had had, for upwards of twenty-five years, a tumor in the left side, which she considered as the remains of a dropsical affection, with which she was afflicted at that time. This swelling sometimes produced great pain, and she supported it with a laced bandage. The medical men tried in vain to find this tumor,—it only appeared when she stood upright. The patient also complained that, within the few previous days, she had experienced in the night an extraordinary degree of agitation, accompanied with beating of the arteries of the head, and a degree of moral irritation she could not account for. She could not then sleep; was perfectly conscious of these feelings, and laughed at them in the morning. The symptoms of catarrh and cough gradually yielded; and, on the 22d of June, no remains of disease were perceptible, except a slight heat of the skin, and increased frequency of the pulse. On the 26th, in the evening, having been up all day, and no longer considering herself a patient, whilst occupied in arranging some things in her wardrobe, on a sudden she was heard to scream out, and in the same moment she fell without a sign of life. Many circumstances prevented the body being examined before the burial; but it was inspected six days after death, and four days after the funeral. The body was then found to be in a state of putrefaction, and gave out an extremely nauseous smell, which was much controlled by aspersions of the chloruret of lime; and they found *(as they might have readily known during the life of the patient,)* that there was really no tumor in the abdomen, the swelling arising from an abdominal hernia. In the chest, they found the pericardium containing two coagula of blood, of about three ounces in weight; and the anterior face of the left ventricle presented, at about an inch from the apex, an oval opening, a quarter of an inch long, and about three lines in breadth: its borders were ragged and torn, and the substance of the heart appeared softer in that part than elsewhere. Within, this opening was covered with a brownish fibrous concretion, mixed with the carniæ columnæ.—*(Revue Medicale,* July.)

8. *Tuberculous Affection of the Brain.*—Dr. OZANAM, of Lyons, records the following case:—A young man, twenty-seven years of age, had, from the month of December, 1821, suffered from a continual pain on the left side of the head. In January, he was seized with a strong epileptic fit, which recurred six times between that period and the 13th of November, when he was admitted into the Hôtel Dieu. He was discharged on the 16th, not having had a renewal of the fits, and appearing otherwise in good health. From the month of December, he lost all the pain in his head, and this amended condition continued nearly the whole of the following year: however, towards the end of last October, he perceived a tumor on his left temple. From that time he became timid, dull, and subject to occasional aberrations of intellect. Although moderate pressure on the part produced no pain, yet he always was apprehensive of its being touched.

On the 11th of December, he was attacked with another violent epileptic paroxysm, and was brought into the Hôtel Dieu, and which was succeeded by a second immediately upon his arrival, leaving him in a condition apparently apoplectic. On the left temple there was a prominent tumor, two inches in circumference, with œdema which extended to the eye-lids; and there was pulsation in the tumor, isochronous with the pulse, and ceasing when pressure was made upon the carotid artery. The tumor was soft, fluctuating, without any change of colour upon the skin, and circumscribed by a hard osseous border. The compression of the carotid appeared to relieve the patient, who then opened his eyes. The patient died on the fifth day after his admission.

On examination after death, the tumor appeared externally shrunk. The cranium, raised cautiously, discovered the membranes highly inflamed; and from the cerebral mass drops of blood exuded after each incision. The left parietal bone was eroded completely for the space of six lines. A tubercle, which had not suppurated, adhered to the cerebral mass, and, raising up the dura mater, had caused a hernia with it and the external periosteum. All that portion of the brain corresponding to the temporal fossa was softened, and, as it were, putrified: it contained six tubercles, of the size of small nuts, very hard, and having in the centre a gravelly yellow kernel. All the viscera of the abdomen and thorax were sound. This tumor externally had all the appearance of an aneurism of the temporal artery.—(*Journal Comp.* August.)

9. *On the Semidecussation of the Optic Nerves.*—In a letter to us from a distinguished philosopher in Germany, the following remarks occur: "I do not understand how it happens that the labours of the Germans, and even of other nations, in comparative anatomy, are so little known in England. Many observations and opinions, which are considered as new in your country, have been long known to us in Germany. In proof of this I may mention, that, in the thirty-fourth number of the London Journal of Science, there is an extract from a memoir on the "*Semidecussation of the Optic Nerves,*" in which the illustrious author, from a pathological appearance he observed, infers a partial crossing of the optic nerves, without appearing to know, not only that many authors, from similar grounds, have come to the same conclusion, but also, that this kind of crosssing had been observed in the eye of the human species by Vicq d'Azyr, Caldani, the brothers Wentzel, and Chiasmon, and by G. R. Treviranus in the eye of the Simia Aygula. *Vide Verm. Schriftin, von G. R. and L. C. Treviranus,* Th. iii. p. 168."—(*Edinburgh Phil. Journal.*)

---

### THERAPEUTICS.

10. *Bad effects of the Ergot.*—M. GERARDIN lately read to th Academie Royale de Medecine an account of certain deleterious consequences resulting from the ergot when employed to facilitate labour:—he stated that in the colonies this substance is looked upon as a sure means of producing abortion, and destroying the life of the fœtus in utero:—he therefore is of opinion, that it ought not to be administered without the greatest caution, and that it ought only to be employed in certain cases of want of action in the uterus during labour, or to expel hydatiform masses developed in its cavity.—(*Revue Medicale,* Sept.)

11. *Hydrocyanic Acid in Tænia.*—M. GALNECHE, of Stettin, records the following case.—A boy, three and a half years old, who was afflicted with the *tænia lata,* was recommended to eat strawberries for two days in as great quantities as he pleases, the author observing that where the presence of this worm is suspected, the strawberry generally produces a discharge of some few portions. The third day, at six in the morning, ℥ss. of castor-oil was given; at half-past six, seven, and half-past seven, he took each time fifteen grs. of the powder of male fern root; and, at eight o'clock, six drachms of castor oil. At half-past eight an abundant serous motion took place, and a quarter of an ell of the worm escaped from the anus; the little patient was then placed upon warm water, into which the worm was suffered to be suspended; it was slightly laid hold of at the orifice of the anus, and rubbed over for the space of four inches, with hydrocyanic acid. It immediately made strong efforts to return into the abdomen, so that it was obliged to be held with some degree of force; it suffered some convulsive motions, and was expelled the length of an ell and a half. At the termination of an hour and half another evacuation produced the remainder of the worm, two ells long; the animal was dead, it terminated in a thread-like extremity, surmounted with a reddish head of the size of a millet seed, armed with a little sucker.—*(Journal der Parkitchen Heilhünde,* June.)

12. *Common Salt to correct the fœtor of Cancerous Sores.*—M. Liaubon relates the case of a female with a cancerous ulcer in the right breast, who suffered extremely from the fœtor of the discharge, which was so intolerable that the persons who assisted in dressing the wound could scarcely remain in the room. M. Liaubon ordered a lotion of salt, dissolved in water, to wash the parts: the disease was not arrested by these means, but the fœtor was entirely destroyed.—*(Journal Medicale de la Gironde.)*

13. *Mugwort a remedy in Epilepsy.*—Dr. Burdach, of Triebel, has discovered that the root of the *Artemesia Vulgaris* is an excellent remedy in epilepsy. He recommends this root to be gathered in autumn, about the middle of October, to dry it in the shade without washing it, and not to powder it before it is required to be used. It must be taken in powder, about a coffee spoonful (or little more than fifty grains) in a little warm beer, and about half an hour before the attack, if that can be ascertained. It produces strong perspiration, during which the patient is to remain in bed. Dr. B. recommends a day's interval between the doses, and relates five cases when perfect cures have been effected by it. Experiments have been made with this root, in the polyclinic school at Berlin, upon ten epileptic patients; the result was, that three were cured in a greater or less space of time, three experienced relief, the attacks being at longer intervals and more feeble; and with the four others no effects were perceptible. These last cases are reported by M. Hufeland.—*(Journal der Praktichen Heilkunde,* April.)

14. *Substitute for Castor Oil.*—M. Hufeland asserts, that by mixing one drop of the oil of Croton with an ounce of syrup of poppy, a preparation is obtained, resembling, in a great degree, the Castor oil, and of which one spoonful produces analogous effects. Many successful experiments have been made with this preparation in the Polyclinic School at Berlin.—*(Ibid.)*

15. *Efficacy of Nitrate of Potass in the treatment of Hemorrhage.*—Dr. Zuccari, after various other remedies had proved unsuccessful, had recourse to *pure* nitrate of potass, and asserts, that it had been followed by continued success. He administered it as follows:—after a small bleeding, which was not always necessary, he mixed nitrate of potass, in the quantity of from four to six drachms, in an ounce of gum-water, two or three table-spoonsfuls of which were taken every hour; and refrigerant drinks, with light soup. The dose of the nitrate has been carried to the extent of an ounce, in the same quantity of the vehicle above mentioned, without producing any inconvenience. The author of the paper adds, that he is about to publish a work upon the subject.—(*Annali Universali,* July 1824.)

---

### STATISTICAL MEDICINE.

16. *Influence of Vaccination upon the Mortality of Berlin.*—M. Casper has published a long paper, containing many curious details relative to the above subject; but we can do no more, at present, than give the result of his investigations.

1. The small-pox formerly carried off from the 12th to the 10th of the population.
2. Formerly at Berlin one out of twelve children born, died of the small-pox; now the deaths from the same cause are 1 in 116.
3. The diseases of children are more common than before the introduction of vaccination, because the number of infants that survive is more considerable.
4. These diseases formerly destroyed 39 children out of every 100; now only 34 in the hundred die of these diseases, so that in the whole 51 children in 100 died formerly, and at present only 43.
5. Generally speaking, 1 inhabitant in 28 used to die annually, now there is only 1 death in 34.—(*Journal Comp.* September.)

---

### MISCELLANEOUS.

17. *New Regulations adopted by the Court of Examiners, Apothecaries' Hall.*—Medical Students are requested to take notice, that in consequence of some recent regulations of the Court of Examiners, it is indispensably necessary for all those who enter themselves as physicians' pupils at the hospitals and dispensaries in London, *and who intend to present themselves for examination,* to appear personally at the Hall, and to bring with them the tickets authorising their attendance on the medical practice of such institutions.

A book has been opened at the beadle's office, for the purpose of registering the date of the tickets, and the time of the Student's appearance with them. Every Student who shall bring his ticket for this purpose previously to the seventh of next month (December) will be entitled to calculate the time of his attendance at the hospital or dispensary from the day of the date of his ticket; but every one who shall come after that time, will only be permitted to calculate the period of such attendance from the day of the date of his appearance at the beadle's office.

Persons whose tickets are dated prior to the 1st of October last, are exempted from the necessity of registering such tickets. No fees to be paid for this registry.

### METEOROLOGICAL JOURNAL,

*From October 20, to November 19, 1824.*

By Messrs. William Harris and Co. 50, Holborn, London.

| | MOON. | Rain gauge | THERM. | | | BAROM. | | DE LUC'S HYG. | | WIND. | | ATMOSPHERIC VARIATIONS. | | |
|---|---|---|---|---|---|---|---|---|---|---|---|---|---|---|
| | | | 9 A.M. | MAX. | MIN. | 9 A.M. | 10P.M. | 9 A.M. | 10P.M. | 9 A.M. | 10P.M. | 9 A.M. | 2 P.M. | 10P.M. |
| Oct. | | | | | | | | | | | | | | |
| 20 | | | 54 | 56 | 47 | 29.94 | 29.98 | 72 | 70 | WSW | SW | Fine | Fine | Cloud. |
| 21 | | | 49 | 57 | 49 | 29.96 | 29.88 | 77 | 76 | SW | SSW | Fog | Fine | |
| 22 | ● | | 50 | 53 | 54 | 29.85 | 29.78 | 79 | 79 | SSE | S | Foggy | | Fine |
| 23 | | | 56 | 61 | 52 | 29.80 | 29.89 | 94 | 79 | S | WSW | Fine | | |
| 24 | | | 56 | 63 | 52 | 29.76 | 29.58 | 80 | 78 | SE | ESE | | | Cloud. |
| 25 | | .03 | 59 | 64 | 53 | 29.50 | 29.50 | 78 | 85 | SW | SW | Cloud. | | Fine |
| 26 | | | 56 | 58 | 49 | 29.19 | 29.27 | 80 | 74 | S | SW | Rain | | Cloud. |
| 27 | | .06 | 51 | 56 | 49 | 29.43 | 29.53 | 74 | 72 | WSW | W | Fine | | |
| 28 | | | 52 | 55 | 50 | 29.57 | 29.57 | 85 | 85 | WSW | WSW | | | |
| 29 | ☽ | | 51 | 54 | 41 | 29.57 | 29.45 | 90 | 80 | SWva. | WNW | Rain | Rain | Cloud. |
| 30 | | .19 | 54 | 57 | 40 | 29.76 | 30.05 | 60 | 70 | WNW | WNW | Cloud. | Fine | Fine |
| 31 | | | 45 | 49 | 45 | 29.90 | 29.68 | 80 | 87 | WSW | W | Rain | Rain | Rain |
| Nov | | | | | | | | | | | | | | |
| 1 | | | 50 | 55 | 54 | 29.55 | 29.52 | 82 | 89 | W | W | Fine | Cloud. | Cloud. |
| 2 | | | 56 | 58 | 49 | 29.63 | 29.44 | 89 | 79 | WSW | W var. | Sho'ry | Fine | Fine |
| 3 | | .34 | 51 | 56 | 42 | 29.73 | 29.66 | 78 | 77 | WSW | WSW | Sl. Fog | | |
| 4 | | | 44 | 47 | 40 | 29.70 | 29.71 | 75 | 70 | WNW | WSW | Fine | | |
| 5 | | | 41 | 46 | 34 | 29.65 | 29.79 | 80 | 70 | WNW | WNW | | | |
| 6 | ○ | | 36 | 45 | 45 | 29.96 | 29.97 | 79 | 72 | WNW | S | Foggy | | |
| 7 | | | 54 | 57 | 54 | 29.85 | 29.60 | 92 | 88 | WSW | S | Rain | Cloud. | |
| 8 | | .22 | 56 | 57 | 43 | 29.58 | 29.53 | 91 | 89 | SW | W | Misty | | Cloud. |
| 9 | | | 44 | 47 | 46 | 29.85 | 29.87 | 83 | 75 | WNW | SW | Fine | Fine | Sho'ry |
| 10 | | | 52 | 56 | 51 | 29.76 | 29.67 | 78 | 80 | W | SWva. | F.&M. | | Cloud. |
| 11 | | | 52 | 56 | 44 | 29.73 | 29.66 | 94 | 94 | WNW | WSW | Rain | Rain | Rain |
| 12 | | .30 | 48 | 55 | 53 | 29.84 | 30.04 | 82 | 74 | WNW | WSW | Fine | Fine | Fine |
| 13 | | | 54 | 55 | 55 | 29.72 | 29.63 | 80 | 86 | WSW | WSW | | Cloud. | Cloud. |
| 14 | ☾ | | 56 | 58 | 40 | 29.47 | 29.60 | 88 | 80 | W var. | W | Rain | | |
| 15 | | | 42 | 46 | 37 | 29.83 | 30.04 | 79 | 74 | W | NW | Foggy | Sho'ry | Fine |
| 16 | | .26 | 41 | 50 | 45 | 30.14 | 29.93 | 75 | 80 | SSW | SW | | Fine | |
| 17 | | | 50 | 54 | 55 | 29.67 | 29.50 | 82 | 87 | SW | WSW | | Cloud. | Cloud. |
| 18 | | | 59 | 58 | 46 | 29.31 | 29.60 | 88 | 84 | SW | SW | Rain | Rain | Cloud. |
| 19 | | .54 | 48 | 50 | 48 | 29.70 | 29.61 | 88 | 95 | SW | NE | Fine | | Rain |

The quantity of rain fallen in the month of October, was 2 inches and 16.100ths.

